# Weakly perturbative imaging of interfacial water with submolecular resolution by atomic force microscopy

**DOI:** 10.1038/s41467-017-02635-5

**Published:** 2018-01-09

**Authors:** Jinbo Peng, Jing Guo, Prokop Hapala, Duanyun Cao, Runze Ma, Bowei Cheng, Limei Xu, Martin Ondráček, Pavel Jelínek, Enge Wang, Ying Jiang

**Affiliations:** 1 International Center for Quantum Materials, School of Physics, Peking University, Beijing, 100871 China; 2 Institute of Physics, The Czech Academy of Sciences, Cukrovarnická 10, 162 00 Prague, Czech Republic; 3 Collaborative Innovation Center of Quantum Matter, Beijing, 100871 China; 4 RCPTM, Palacky University, Šlechtitelů 27, 783 71 Olomouc, Czech Republic; 5 CAS Center for Excellence in Topological Quantum Computation, University of Chinese Academy of Sciences, Beijing, 100190 China

## Abstract

Scanning probe microscopy has been extensively applied to probe interfacial water in many interdisciplinary fields but the disturbance of the probes on the hydrogen-bonding structure of water has remained an intractable problem. Here, we report submolecular-resolution imaging of the water clusters on a NaCl(001) surface within the nearly noninvasive region by a qPlus-based noncontact atomic force microscopy. Comparison with theoretical simulations reveals that the key lies in probing the weak high-order electrostatic force between the quadrupole-like CO-terminated tip and the polar water molecules at large tip–water distances. This interaction allows the imaging and structural determination of the weakly bonded water clusters and even of their metastable states with negligible disturbance. This work may open an avenue for studying the intrinsic structure and dynamics of ice or water on surfaces, ion hydration, and biological water with atomic precision.

## Abstract (web summary)

Scanning probe microscopy has been extensively applied to probe interfacial water but the probes tend to disturb the structure of water easily. Here, the authors report submolecular-resolution imaging of water clusters within the nearly non-invasive region by qPlus noncontact atomic force microscopy.

## Introduction

Water–solid interactions are of broad importance in many basic and applied fields, ranging from surface science to materials science and even bioscience^1–4^. In particular, resolving the hydrogen-bonding (H-bonding) structure of interfacial water is crucial for understanding many extraordinary physical and chemical properties of water/solid interfaces. To date, scanning probe microscopy (SPM), including scanning tunneling microscopy (STM)^5–16^ and atomic force microscopy (AFM)^17–23^, has been an ideal tool to visualize the microscopic structure and dynamics of water at solid surfaces. However, an intrinsic problem of SPM is that all the probes inevitably induce perturbation to the fragile water structure, due to the excitation of the tunneling electrons and the tip–water interaction forces, especially under the close-imaging condition applied in order to achieve ultrahigh spatial resolution. This limitation makes SPM fall short compared with noninvasive spectroscopic methods such as optical spectroscopy, neutron scattering and nuclear magnetic resonance.

Recent advances in qPlus-based^24^ noncontact AFM (nc-AFM) show the ability to achieve superior resolution and sensitivity of single molecules/atoms, such as identifying the chemical structure and intermolecular interaction^25–29^, determining the bond order^30^ and chemical reaction products^31^, imaging the charge distribution within a molecule^32^, measuring the force needed to move an atom^33^, and even revealing the internal structure of metal clusters^34^. Unfortunately, the atomic resolution of organic molecules is typically achieved at the very small tip–molecule separation, where the short-range Pauli repulsion force is dominant^25, 35^. The tip–molecule interaction in this range is quite strong such that significant relaxation of the tip apex is induced^35^. Considering that H bonds are much weaker than covalent bonds, the water structure may be easily disturbed at small tip heights^21^. At large tip heights where only the long-range van der Waals and electrostatic forces are detectable, the resolution is usually quite poor for weakly polarized molecules. However, in contrary to the weakly polarized aromatic molecules, the water molecule has a strong internal dipole moment. Therefore, the imaging mechanism driven by the electrostatic force greatly relies on the detailed charge nature of the tip apex^36, 37^.

Herein, we report the submolecular-resolution imaging of water nanoclusters on a Au-supported NaCl(001) film by probing the high-order electrostatic force using a qPlus-based nc-AFM. The AFM images of the water tetramers taken with a CO-terminated tip at large tip–water distance show prominent internal features, which resemble the electrostatic potential distribution within the cyclic tetramer. Comparison with the theoretical simulations reveals that such a high resolution originates from the electrostatic force acting between the quadrupole-like CO-tip and the strongly polar water molecules. In contrast, the results obtained with a monopole-like Cl-tip show much poorer resolution at large tip heights, arising from the different decay behaviors of the tip–water electrostatic interaction and the different charge distribution at the tip apex. Strikingly, the multipole electrostatic force between the CO-tip and water is rather weak, thus allowing precise structural determination of the weakly bonded water clusters and even their metastable states with negligible disturbance.

## Results

### AFM images of two degenerate water tetramers with a CO-terminated tip

**Fig. 1:**
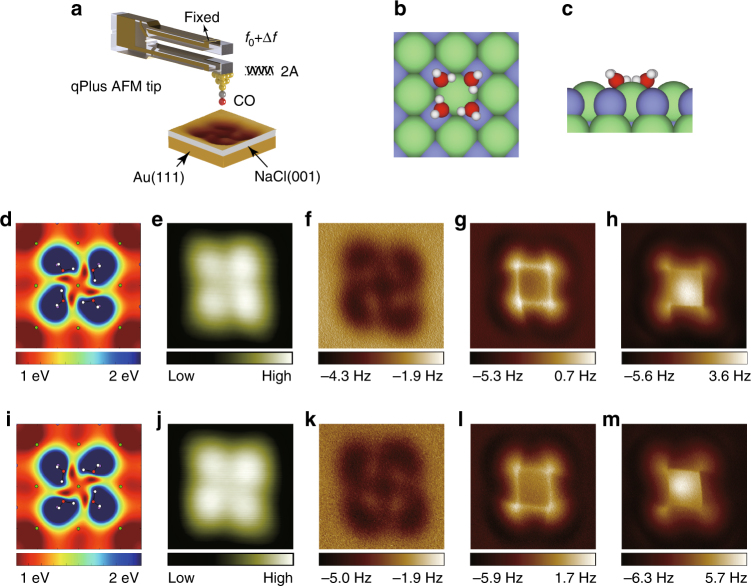
Experimental set-up and AFM images of two degenerate water tetramers with a CO-terminated tip. **a** Schematic of a qPlus-based nc-AFM with a CO-tip. The cantilever oscillates at an amplitude of A and the tip-sample force-induced frequency shift of the cantilever from its natural resonance frequency (*f*_0_) is Δ*f*. **b**, **c** Top and side view of the water tetramer adsorbed on the NaCl(001) surface, respectively. H, O, Cl, and Na atoms are denoted as white, red, green, and purple spheres, respectively. **d**–**h**, **i**–**m** Water tetramers with clockwise and anticlockwise H-bonded loops, respectively. **d**, **i** Calculated electrostatic potential map of the water tetramers in a plane 60 pm above the outermost H atom. **e**, **j** Constant-current STM images acquired at (100 mV, 20 pA) and (100 mV, 30 pA), respectively. **f**, **k**, **g**, **l**, **h**, **m** Experimental Δ*f* images recorded at the tip heights of 100 pm, 10 pm, −40 pm, respectively. The tip height is referenced to the STM set point on the NaCl surface (100 mV, 50 pA). The oscillation amplitude is 100 pm. The STM/AFM imaging was done on two different tetramers with two different CO-tips. The size of the images is 1.2 nm × 1.2 nm

The experimental setup is schematically shown in Fig. 1a, where the tip apex is functionalized with a CO molecule (see “Methods” section). Water tetramers were constructed by assembling four individual H_2_O monomers on the NaCl(001) surface at 5 K. Our previous work reveals that each water molecule acts as a single H-bond donor and single H-bond acceptor resulting in a cyclic tetramer (Fig. 1b), whereas the other four free OH bonds point obliquely upward away from the surface (Fig. 1c)^12^. In fact, the cyclic water tetramer may form two degenerate chiral H-bonded loops, which are, respectively, displayed in Fig. 1d, i, with the calculated Hartree potential superimposed. Figure 1e, j is the constant-current STM images of the two degenerate tetramers with a CO-tip, where no obvious chiralities can be discerned. Figure 1f–h, k–m is constant-height Δ*f* images with the CO-tip at three different tip heights. At a large tip height, the two tetramers were imaged as four “ear-like” depressions with distinct chirality (Fig. 1f, k, and the corresponding rescaled images in Supplementary Fig. 1), which closely resemble their electrostatic potential (Fig. 1d, i). As the tip height decreased, the H-bonded loop was visualized as a bright square (Fig. 1g, l). When further approaching the tip, besides the sharpening of the square lines, contrast inversion was also observed at the center of the tetramer (Fig. 1h, m). Interestingly, from Fig. 1g, h, l, m, it is evident that the chiral contrast almost vanishes at small tip heights.

### Role of electrostatics in the high-resolution AFM imaging of a water tetramer

**Fig. 2:**
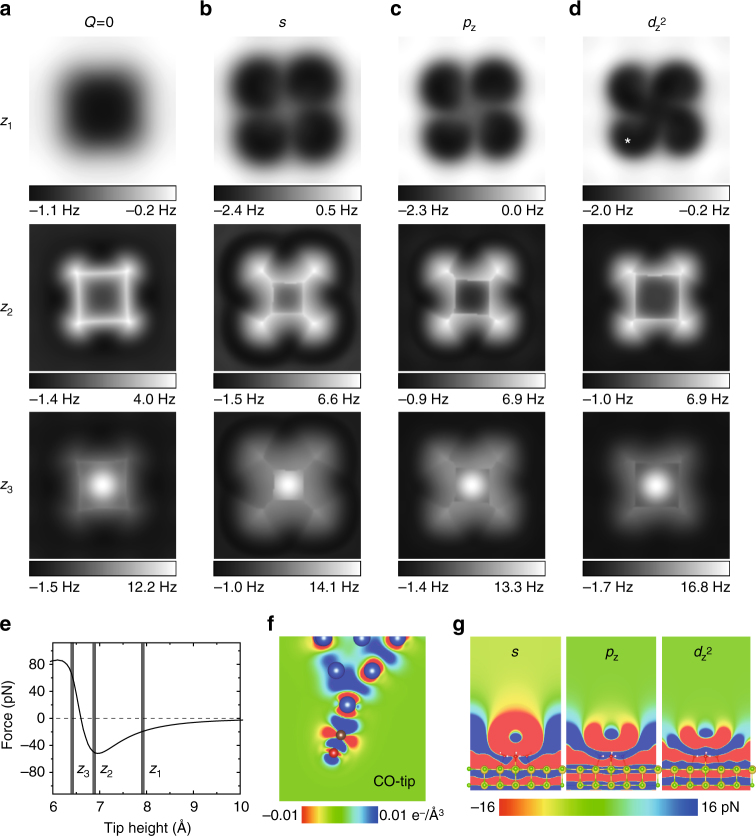
The role of electrostatics in the high-resolution AFM imaging of a water tetramer. **a***–***d** Simulated AFM images of a water tetramer with neutral, *s*, *p*_z_ and ${d}_{{\mathrm{z}}^2}$ tip models, respectively (*k* = 0.5 N m^−1^, *Q *= −0.2 e). The first, second, and third rows correspond to the images acquired at the tip heights of about *z*_1_ = 7.9 Å, *z*_2_ = 6.8 Å and *z*_3_ = 6.4 Å, respectively. For a better comparison, we had chosen similar simulation images by subtracting a small offset of tip height between different tips. The tip height is defined as the distance between the outmost metal atom of the tip and the upward H atom of the water tetramer. The oscillation amplitude of all the simulated Δ*f* images is 100 pm. The size of the images is 1.2 nm × 1.2 nm. **e** Simulated force curve of the water tetramer taken with the ${d}_{{\mathrm{z}}^2}$ tip, where the three tip heights (*z*_1_, *z*_2_, and *z*_3_) are denoted. The tip position is indicated by a star in **d**. **f** Charge distribution of the CO-tip from DFT calculations. **g** Maps of calculated vertical electrostatic forces between the sample and different tip models (*s*, *p*_z_ and ${d}_{{\mathrm{z}}^2}$) computed by convolution of Hartree potential of sample and model charge distribution on the tip^39^

It is very unusual to obtain submolecular contrast (Fig. 1f, k) at large tip heights, where the long-range force dominates the tip–water interaction. To understand the imaging mechanism, we used a molecular mechanics tip model (see “Methods” section) to simulate the AFM images (Fig. 2a–d). We analyze the AFM contrast at different tip heights *z*_1_, *z*_2_, and *z*_3_ as denoted in Fig. 2e. The simulated Δ*f* images of an anticlockwise tetramer with the neutral tip model (Fig. 2a, *z*_2_ and *z*_3_) agree well with the experimental results at small tip heights (Fig. 1l, m). Detailed analysis (see Supplementary Fig. 2 for details) reveals that the sharp lines and the contrast inversion both result from the Pauli repulsion and the consequent lateral relaxation of the CO molecule at the tip apex, similar to previous studies of aromatic molecules^25, 38^. The sharp edges observed in AFM images should not be automatically related to the presence of interatomic bonds, instead they represent ridges of the potential energy landscape experienced by the functionalized probe^35, 39^.

However, the simulation for the neutral tip at the large tip height (Fig. 2a, *z*_1_) fails to reproduce the internal chiral structure of tetramer (Fig. 1k). Figure 2b–d compares simulated Δ*f* images using monopole (*s*), dipole (*p*_z_), and quadrupole (${d}_{{\mathrm{z}}^2}$) tip models, respectively (Supplementary Fig. 3). The simulated images with the monopole and dipole tips at the large tip height (Fig. 2b, c, *z*_1_) show very little chirality. In contrast, the “ear-like” chiral features in Fig. 1k can be perfectly reproduced with the quadrupole tip (Fig. 2d, *z*_1_), which also yields good agreement with the experimental images at the small tip heights (Fig. 2d, *z*_2_ and *z*_3_). We note that the simulation results are insensitive to the stiffness (*k*) of the tip, but greatly rely on the effective charge density (*Q*) (Supplementary Fig. 4). In fact, the quadrupole nature of the CO-tip can be verified from the plot of charge density difference calculated by density functional theory (DFT) (Fig. 2f). It is a result of charge redistribution between the adsorbed CO and metal tip^37^.

The variation of the AFM contrast using different tip models can be understood from the analysis of calculated electrostatic forces acting between the sample and the given tip model. In Fig. 2g, we plot *xz*-cut planes of vertical electrostatic force, which show significantly different shapes and decay behaviors for different charged tip models. Indeed, from the simulated electrostatic force curves over the water tetramer, we can see that the electrostatic force between the quadrupole tip and water decays much faster than the others as increasing the tip height (Supplementary Fig. 5 and Supplementary Table 1). Such a difference in the decay behavior can be also seen from the experimental force curves acquired with CO-tip (quadrupole) and Cl-tip (monopole) (Supplementary Fig. 5 and Supplementary Table 1). The long-range electrostatic force between the monopole tip and water only creates a large attractive background in the AFM images, thus hindering submolecular contrast.

Furthermore, we note that the lateral potential profile of CO-tip apex resembles well the “Mexican hat” wavelet (see Supplementary Fig. 6), which acts as an internal high-pass filter (actually Laplace filter, see also Supplementary Fig. 3). A tip with such kind of charge distribution can filter out the smoothly varying force components and becomes more sensitive to the atomic details. To the best of our knowledge, such a high-resolution image of electrostatic force has never been achieved for aromatic molecules (such as pentacene, cephalandole A, phthalocyanine, etc.) with the CO-tip at far tip–sample distances. The main reason is that the water molecule has a much larger dipole moment than those aromatic molecules. In such a case, the multipole charge distribution of the CO-tip and the related electrostatic force should be taken into consideration to explain the improved resolution.

### AFM images of two degenerate water tetramers with a Cl-terminated tip

**Fig. 3:**
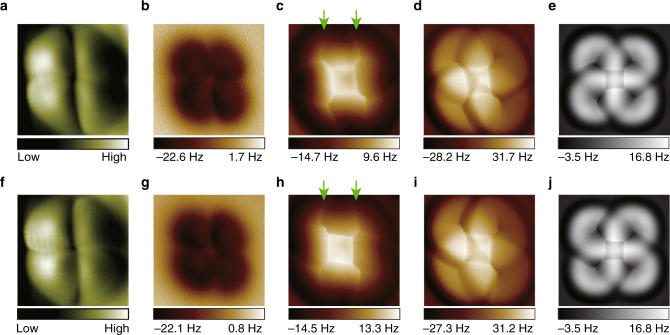
Experimental and simulated AFM images of two degenerate water tetramers with a Cl-terminated tip. **a**, **f** Derivative STM images of the water tetramers with clockwise and anticlockwise hydrogen-bonded loops, respectively. Set point: 10 mV and 50 pA. **b**–**d**, **g**–**i** Corresponding Δ*f* images. All the images were obtained on the same tetramer (switched) with the same Cl-tip. The tip heights are 30 pm (**b**, **g**), −120 pm (**c**, **h**), −120 pm (**d**, **i**). The oscillation amplitudes are 40 pm (**b**, **g**), 100 pm (**c**, **h**), 40 pm (**d**, **i**). The “fork-like” features are denoted by two green arrows in **c** and **h**. It is noted that the smallest tip heights for the AFM imaging here are at least 20 pm larger than those for the perturbative chirality switching in ref. ^15^. **e**, **j** Simulated Δ*f* images with the oscillation amplitudes of 40 pm, which were obtained with a monopole (s) tip (*k *= 0.5 N m^−1^, *Q *= −0.25 e). The size of the images is 1.2 nm × 1.2 nm

In order to verify the proposed imaging mechanism above, we functionalized the tip apex with a Cl atom. According to our previous DFT simulations^15, 40^, the Cl atom is negatively charged with about 0.3–0.4 e when attached to the metal tip, acting as a monopole tip (see Supplementary Fig. 6). Figure 3a, f displays the derivative STM images of the two degenerate tetramers recorded with a Cl-tip. Figure 3b, g is the constant-height Δ*f* images at a large tip height, showing negligible chirality. This is consistent with the AFM simulation using the monopole tip (Fig. 2b, *z*_1_), revealing the low sensitivity of monopole-like probe charges for high-resolution mapping of complex electrostatic fields. At smaller tip heights, the Δ*f* images (Fig. 3c, h) show prominent sharp squares and “fork-like” features at the periphery (see the green arrows), also agreeing well with the simulation (Fig. 2b, *z*_2_). We note that the central sharp squares show a clear counterclockwise (Fig. 3c)/clockwise (Fig. 3h) rotation, arising from the tip relaxation induced by the strong electrostatic interaction between the Cl-tip and water molecules. Such a rotation is absent for the AFM images acquired by the CO-tip due to the much weaker electrostatic interaction (Fig. 1g, i).

When using a smaller oscillation amplitude, the Δ*f* images change remarkably, showing bright helical structures with distinct chirality (Fig. 3d, i), similar to the chiral depression observed with the CO-tip at the large tip height (Fig. 1f, k) (for the effect of oscillation amplitude, see Supplementary Fig. 7). From the simulations (Fig. 3e, j), we found that those chiral structures obtained with the Cl-tip arise from the pronounced tip relaxation at close tip–water distances, which is determined by the complex interplay between the Pauli and the electrostatic interaction (see Supplementary Fig. 2 and Supplementary Fig. 8). In contrast to the large relaxation with the Cl-tip, we found that the quadrupole tip shows only negligible lateral relaxation at the large tip height, where the submolecular electrostatic potential mapping is obtained (Supplementary Fig. 8), suggesting that the tip–water interaction force is very small such that the disturbance of the CO-tip on the water structure should be minimal in this range. This may open up the possibility of probing weakly bonded water clusters other than the rigid tetramers.

### Submolecular-resolution AFM images of weakly bonded water clusters with a CO-tip

**Fig. 4:**
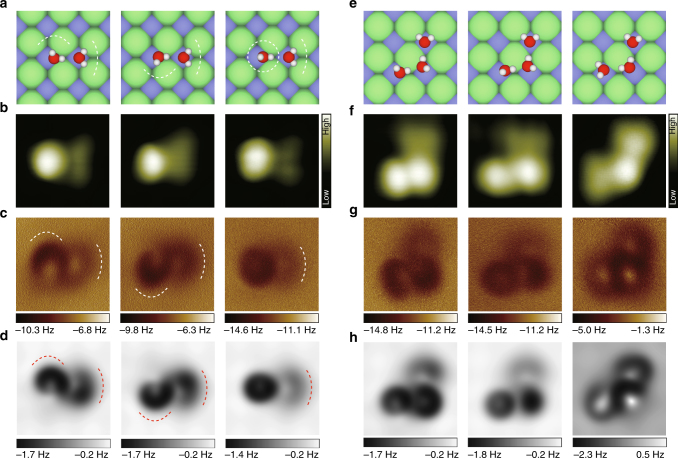
Submolecular-resolution AFM images of weakly bonded water clusters with a CO-tip. **a**–**d** Geometric structures, experimental STM images, experimental and simulated Δ*f* images of two water dimers, respectively. The crooked depressions in the AFM images are highlighted by dashed lines in **a**, **c**, and **d**. Set point of **b**: 100 mV and 50 pA, 30 pA, 25 pA (from left to right), respectively. The tip height of **c** is 100 pm, 100 pm, and 130 pm (from left to right), respectively. **e**–**h** Geometric structures, experimental STM images, experimental and simulated Δ*f* images of three water trimers, respectively. Set point of **f**: 100 mV and 25 pA, 20 pA, 15 pA (from left to right), respectively. The tip height of **g** is 130 pm, 130 pm and 110 pm (from left to right), respectively. All the oscillation amplitudes of experimental and simulated images are 100 pm. All the simulations were done with a quadrupole (${d}_{{\mathrm{z}}^2}$) tip (*k *= 0.5 N m^−1^, *Q *= −0.2 e). The images of the first two dimers were obtained with the same CO-tip, while the third dimer was measured with a different CO-tip at a different site. The three trimers were imaged at different sites with different CO-tips. Size of the images: 1.2 nm × 1.2 nm

To confirm this possibility, we investigated fragile water structures such as dimers and trimers, which were easily disturbed under close-imaging conditions. Figure 4a–d is the geometric structures, experimental STM images, experimental and simulated Δ*f* images of three water dimers at large tip heights, respectively. The STM images show essentially the same features without any internal structures. In contrast, the depression features in AFM images directly reflect the distribution of electrostatic potential in the water dimers (Supplementary Fig. 9). It is worthy to be noted that the crooked depressions in the AFM images are actually correlated with the position of the H atoms (see the dashed lines in Fig. 4a, c, d), which can help us identify the detailed configuration of various water clusters with unprecedented precision. It is striking that the AFM imaging can readily distinguish the subtle difference of the O–H tilting in the water dimers. At small tip heights, we found that the water dimer can be easily switched by the tip (see Supplementary Fig. 10 for details), with an energy barrier only ~65 meV according to DFT calculations (Supplementary Fig. 10). In order to estimate the perturbation of the probes during the submolecular-resolution AFM imaging, we extracted the tip–water interaction energy from the force curves, which can be as small as 40–50 meV (Supplementary Fig. 11).

Water trimers are even more unstable than the dimers since they can have many metastable states, but we are still able to image the electrostatic potential of various water trimers with submolecular resolution (Fig. 4e–h and Supplementary Fig. 9). In combination with the simulations, their atomic configurations can be unambiguously determined. The calculated adsorption energies of those metastable water trimers are very close (Supplementary Table 3), such that they can rapidly fluctuate among different states in the presence of external perturbation (Supplementary Fig. 10). The ability of discerning them suggests that our probe is indeed nearly noninvasive.

## Discussion

We would like to emphasize here that the observed submolecular contrasts in the AFM images of water tetramers, dimers, trimers, and other clusters solely arise from the orientation of the water molecules, instead of different adsorption sites of water clusters on the NaCl surface. In order to clarify this issue, we have performed detailed AFM simulations on the frozen water structures, where the NaCl substrates were removed. It turns out that all the features keep exactly the same no matter with or without NaCl substrate, indicating the negligible role of the NaCl substrate in the AFM imaging. In addition, we fixed the oxygen atoms and only change the position of the H atoms during the simulation, which confirms that the AFM contrasts originate only from the position of the H atoms.

It is worthy to recall that the Cl-tip can also obtain submolecular-resolution imaging of the electrostatic potential of water tetramer by using small oscillation amplitudes (Fig. 3d, i). However, such a resolution is only achieved at small tip–water separation where the electrostatic and Pauli force becomes strong enough to induce significant relaxation of the tip apex. Any attempts to enter into this region can easily disturb the weakly bonded water clusters such as the water dimers, trimers, and bilayer ice clusters (Supplementary Fig. 12). Therefore, the high-order electrostatic force between the CO-tip and the water is critical since it yields submolecular resolution at relatively large tip–water separations, where the electrostatic force and other force components are still rather weak, thus avoiding the disturbance of the tip on the water molecules.

In conclusion, the weakly perturbative imaging achieved in this work defeats the longstanding limitation in the SPM studies of water at surfaces, and may open up an era of studying the intrinsic or “hidden” structures of ice/water on surfaces, ion hydration, and biological water with atomic precision. The submolecular-resolution AFM images of water obtained by CO-tip not only provide the spatial information of electrostatics, but also allow us to determine the detailed H-bonding structure including the position of the H atoms, which is crucial for the understanding of H-bonding interaction and dynamics of water. Furthermore, those results shed light on the mechanism of high-resolution AFM images, highlighting the key roles of the complex charge distribution of the tip apex in the imaging of the polar molecules. In principle, this technique can be easily generalized to other polar systems by carefully engineering the charge nature of the tip apex.

## Methods

### STM/AFM experiments

All the experiments were performed with a combined nc-AFM/STM system (Createc, Germany) at 5 K using a qPlus sensor equipped with a W tip (spring constant *k*_0_ ≈ 1800 N m^−1^, resonance frequency *f*_0_ = 23.7 kHz, and quality factor *Q *≈ 80,000). The NaCl(001) bilayer film was obtained by thermally evaporating NaCl crystals onto a clean Au(111) surface at room temperature. The ultrapure H_2_O (Sigma-Aldrich, deuterium-depleted) was used and further purified under vacuum by several freeze-and-pump cycles to remove remaining impurities. The H_2_O molecules were dosed in situ onto the sample surface at 5 K through a dosing tube. All of the frequency shift (Δ*f*) images were obtained in constant-height mode at 5 K with Cl- or CO-terminated tips. The preparation of the Cl-tip was the same as in ref. ^15^. The CO-tip was obtained by positioning the tip over a CO molecule adsorbed on the NaCl film at a set point of 100 mV and 20 pA, followed by increasing the bias voltage to 200 mV. The controllable manipulation of water monomers to construct water tetramers was achieved with the Cl-terminated tip at the set point: *V* = 10 mV, *I* = 150 pA. The bias voltage refers to the sample voltage with respect to the tip. The contrast of the images does not change at different scanning directions, so we did not explicitly denote the scanning directions unless it is necessary.

### Simulations of AFM images

The Δ*f* images were simulated with a molecular mechanics model, including the electrostatic force, based on the methods described in refs. ^35^ and ^39^. We used the following parameters of the flexible probe-particle tip model: the effective lateral stiffness *k* = 0.5 N m^−1^ and effective atomic radius *R*_c_ = 1.66 Å. In order to extract the effect of electrostatics more clearly and to make *z*-distance directly comparable, we used the same stiffness and atomic radius to simulate AFM images acquired with CO and Cl-terminated tips. Noteworthy, the simulated Δ*f* images using different atomic radius of the probe particle to mimic CO (*R*_c_ = 1.66 Å) and Cl (*R*_c_ = 1.95 Å) tip-apex models with the same effective charges display essentially the same features. The input electrostatic potentials of water tetramer (using previously optimized atomic structure from ref. ^12^) and other water clusters were obtained by DFT calculation using the VASP code with a plane-wave cutoff 600 eV and 550 eV, respectively. Parameters of Lennard Jones pairwise potentials for all elements are listed in Supplementary Table 2.

### DFT calculations

DFT calculations were performed using the Vienna ab-initio simulation package (VASP^41^). In the calculations, we used the Projector augmented wave method (PAW^42^) with PBE functional^43^. Van der Waals corrections for dispersion forces were considered using the van der Waals density functional scheme with the optB88-vdW method^44^. Similar to ref. ^12^, we used a bilayer NaCl(001) slab separated by a vacuum thicker than 20 Å and the bottom layer of the NaCl was fixed with a bulk lattice constant of 5.665 Å. Supercells with Monkhorst–Pack k-point meshes of spacing denser than $2\pi \times 0.042$ Å^–1^ and a plane-wave cutoff 550 eV were used. The geometry optimizations were run with the energy criterion of 5 × 10^−5^ eV and the adsorption energy was calculated by subtracting the total energy of the *n*H_2_O/NaCl(001) structure from the sum of the energies of the relaxed bare NaCl(001) substrate and n isolated water molecules in gas phase:

1 $$E_{\mathrm{ads}} = E \left[ \left( {\mathrm{NaCl}} ({001}) \right)_{\mathrm{relaxed}} \right] + {n} \times E \left[ \left( {\mathrm{H}}_{2}{\mathrm{O}} \right)_{\mathrm{gas}} \right] - E \left[ \left( {\mathrm{NaCl}} \left( {001} \right) + n {\mathrm{H}}_{2}{\mathrm{O}} \right)_{\mathrm{relaxed}} \right]$$

Energy barrier of water dimer was determined using the climbing image nudged elastic band (cNEB) method^45^ with the force criterion of 0.02 eV Å^−1^

### Data availability

The data that support the findings of this study are available from the corresponding authors on request.

## Electronic supplementary material

Supplementary Information
