# Supplementary material for: Weakly perturbative imaging of interfacial water with submolecular resolution by atomic force microscopy
Source: Nat Commun. 2018 Jan 9;9:122. doi: 10.1038/s41467-017-02635-5 (PMC5760619; doi:10.1038/s41467-017-02635-5)
Supplement: Supplementary file 1 — Supplementary Information [file 41467_2017_2635_MOESM1_ESM.pdf]

Supplementary Note 1. Rescaled Δf images of water tetramers

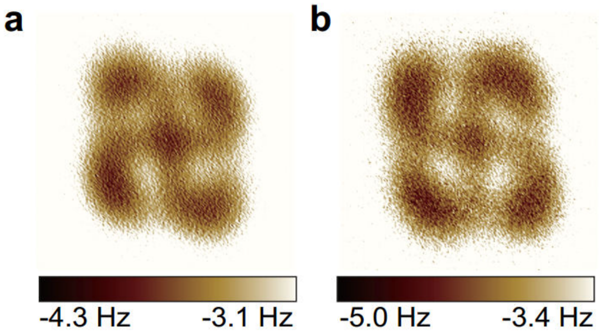

Supplementary Figure 1. Rescaled Δf images of Fig. 1f (a) and k (b). The two different chiralities of the tetramers can be seen more clearly.

Supplementary Note 2. Submolecular contrasts in Δf images at small tip heights

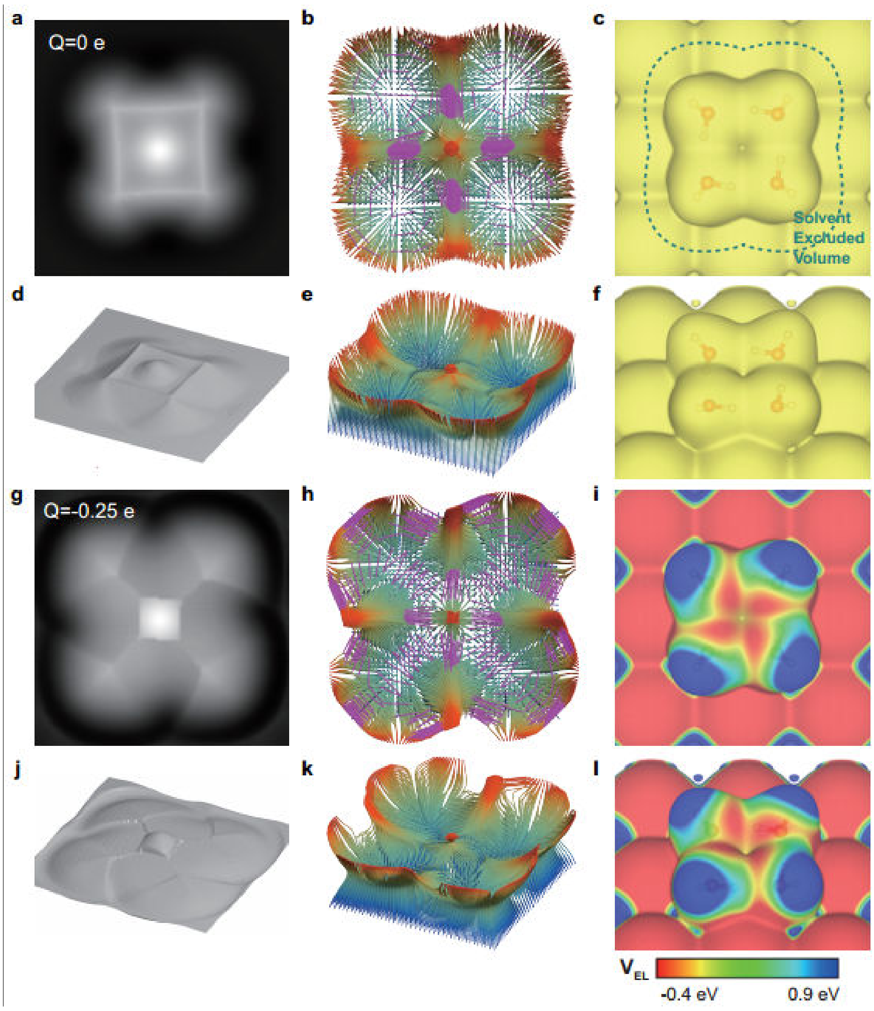

Supplementary Figure 2. Simulated Δf images, probe-particle trajectories, and Pauli and electrostatic potentials for water tetramer. (**a-f**) Neutral tip. (**g-l**) Negatively charged monopole tip (Q=-0.25 e). (**a** and **g**) Simulated Δf images. (**d** and **j**) 3D relief generated from the simulated Δf image. (**b**,**h**) and (**e**,**k**) Top and 3D view of probe-particle trajectories when approaching the tip to the surface (from blue to red). Purple lines denote branching of trajectories. More specifically, they were plotted when two neighboring trajectories diverge by more than 0.4 Å. (**c** and **f**) Isosurfaces of the total electron density which probe-particle cannot penetrate due to the Pauli repulsion. (**i** and **l**) Electrostatic potential mapped on top of the electron density isosurface (**c** and **f**). The presence of the strong electrostatic fields varies trajectories of charged probe particle. The trajectory modification introduces additional branching points (**h** and **k**), giving rise to the fork-like features in the Δf images (**g** and **j**).

The sharp lines in the Δf images emerge from branching of probe-particle trajectories over saddle points of the total tip-sample interaction potential at small tip-water separations as discussed in Supplementary ref. 1. In the case of non-planar and strongly polarized system, such as water clusters, it leads to even more intriguing and unintuitive results, which deserve detailed discussion. The total interaction potential between the functionalized tip and the water molecules adsorbed on surface consists of Pauli repulsion, London dispersion and electrostatic interaction.

In the case of CO-tip, the image contrast can be fully understood by simulations (Supplementary Fig. 2a-f) that consider just the former two components of the potential (Pauli repulsion and London dispersion). This assumption can be justified by a small charge presented on the CO-tip (see Supplementary Fig. 6). Characteristic sharp square lines appear between the upward H atoms as a result of the saddles in the Pauli repulsion, which are visible also in a contour of the total electron density of the cluster (see Supplementary Fig. 2c and f). Due to the finite van der Waals radius of the probe particle (see Supplementary Fig. 6), it moves around on slightly larger surface as described by the concept of “solvent excluded volume” introduced in biochemistry[2]. The potential saddles lead to branching of the probe particle trajectories (Supplementary Fig. 1b and e), which gives rise to the sharp square in the Δf images (Supplementary Fig. 2a and d). The center of the sharp square exhibits contrast inversion at very close tip-sample distance (see Supplementary Fig. 1h and m) as the probe

particle is locked in the center of the square and further relaxation is prevented.

On the contrary, the image contrast acquired with the Cl-tip is strongly affected by the electrostatic field of the water cluster, leading to very different features at small tip-sample distance, which can be also reproduced by our simulations using a monopole tip (see Supplementary Fig. 2g and j, Fig. 3e and j). Based on these simulations, we can rationalize the origin of two main differences compared with the CO-tip case: (i) the shrinking of central square and (ii) appearance of additional fork-like features at the periphery (large amplitude, Fig. 3c and h) and chiral ear-like rings (small amplitude, Fig. 3d and i). All these features can be ultimately tracked down to a map of electrostatic potential (Supplementary Fig. 2i and l) overlaid on top of a contour of total electron density (or Pauli repulsion) along which the probe particle slides upon tip approaching. In the case of Cl-tip, the presence of the electrostatic field above the water tetramer makes the relaxation of probe particle more complicated. The probe particle (Cl ion) is repelled from negatively charged center toward positively charged H atoms, but then it suddenly slips off due to the Pauli repulsion over protruding H atoms and the restoring spring force of the tip. This sudden slip-off leads to additional branching of the probe particle trajectories ultimately manifested as discontinuity of Δf signal measured on different sides of branching line. Thus, it gives rise to the sharp fork-like features and the chiral ear-like rings in the Δf images. The exact position of branching lines is very sensitive to the detailed force balance between electrostatic and other forces (Pauli repulsion, restoring spring force). Therefore, the Δf images obtained with Cl-tip at small tip heights contain some information of the electrostatic field, which is strongly entangled with other force fields.

Supplementary Note 3. The definition of charged tip models

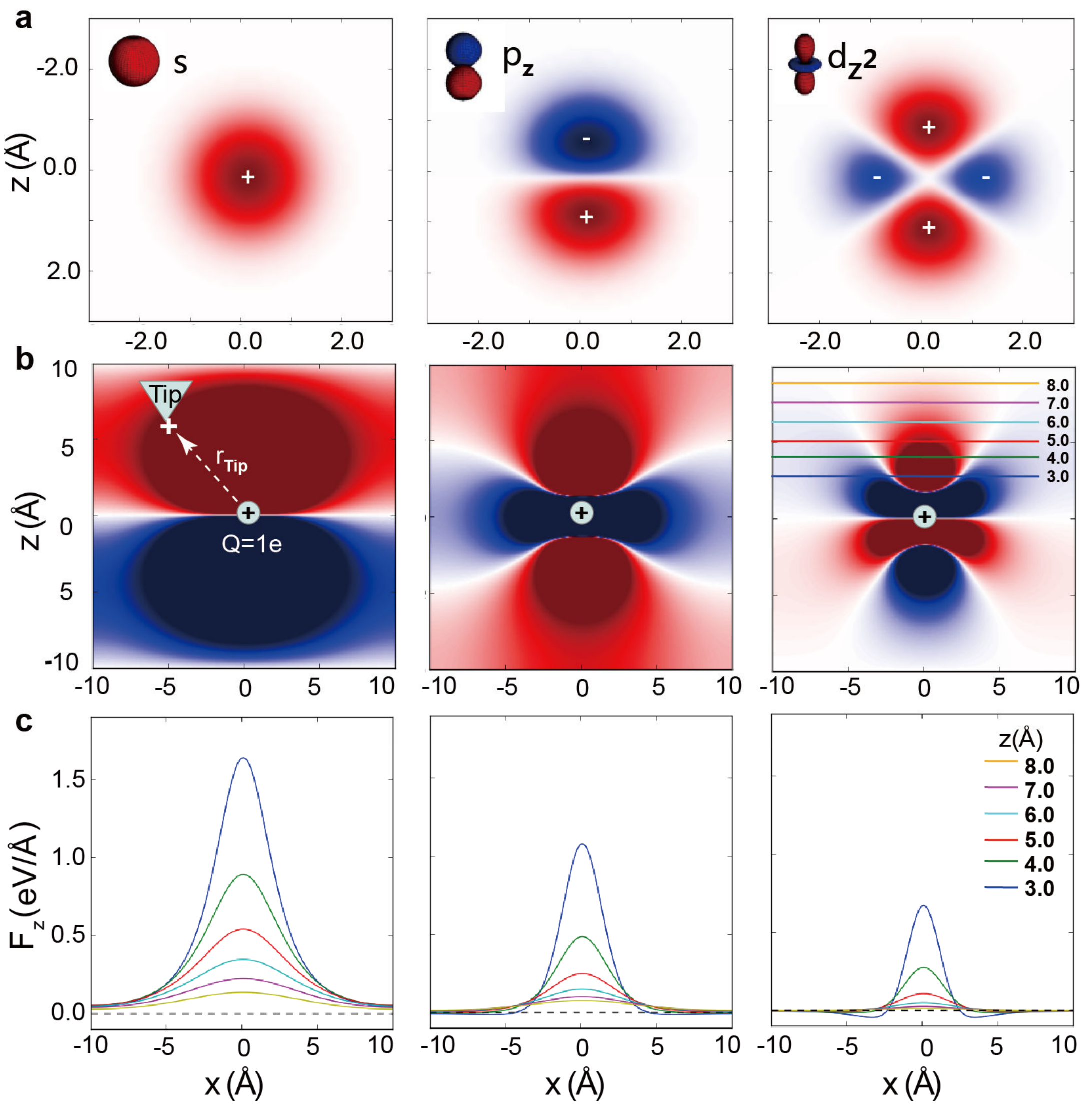

Supplementary Figure 3. Charge distribution and electrostatic force of different charged tip models. (**a**) xz-cut of the charge distribution of monopole (s-like), dipole (p_z) and quadrupole (d_{z^2}), respectively. Detailed description of formulas is given below. We used the smearing width (effective radius) σ = 0.7 Å in all simulations presented in this work. (**b**) xz-cut of the vertical electrostatic force (F_z) between a point charge (Q=1 e, as shown in the center) and s, p_z, d_{z^2} tips which move around the point charge. (**c**) Line profile along x-axis of the vertical electrostatic force F_z at different z distances (as indicated in **b**).

Here, we give specific formulas that define charge distribution on the tip for the

multipole tip models. We have discussed three different models in our present paper: monopole (s), dipole (p_z) and quadrupole (d_{z^2}) (see Supplementary Fig. 3a). A general formula for the spatial distribution of charge density corresponding to a multipolar tip can be written as

$$\rho(x, y, z) = QR_\sigma(r)\phi(x, y, z),$$

where

$$r = \sqrt{x^2 + y^2 + z^2},$$

the function R_σ is a normalized three-dimensional Gaussian that defines the radial part of the charge density:

$$R_\sigma(r) = \frac{e^{-\frac{r^2}{2\sigma^2}}}{(\sqrt{2\pi}\sigma)^3}$$

and the angular part $\phi(x, y, z)$, specific for the multipole in question, is

$$\phi_s(x, y, z) = 1,$$

$$\phi_{p_z}(x, y, z) = z/\sigma,$$

$$\phi_{d_{z^2}}(x, y, z) = \frac{2z^2 - x^2 - y^2}{4\sigma^2}.$$

There are two parameters to be chosen for each of these model distributions: The smearing width (effective radius) σ and an overall multiplicative factor Q. In this paper, we adopt value of σ = 0.7 Å. The normalization of the functions R_σ, ϕ_s, ϕ_{p_z}, and $\phi_{d_{z^2}}$ was chosen so that

$$\int \rho_s(x, y, z)\, dx\, dy\, dz = Q,$$

$$\int z\rho_{p_z}(x, y, z)\, dx\, dy\, dz = Q\sigma,$$

$$\int z^2\rho_{d_{z^2}}(x, y, z)\, dx\, dy\, dz = Q\sigma^2.$$

This choice gives a straightforward interpretation of the factor Q. For a monopole, it is simply the total charge. For a dipole and quadrupole, $Q\sigma$ and $Q\sigma^2$, respectively, give its magnitude.

From the definition of charged tip models, the charge density of a quadrupole is

$$\rho_{d_{z^2}}(x, y, z) = Q\frac{(2z^2 - x^2 - y^2)}{4\sigma^2}R_\sigma(r)$$

Equivalently, it can be written as

$$\rho_{d_{z^2}}(x,y,z) = \frac{Q\sigma^2}{4}\left(2\frac{\partial^2}{\partial z^2} - \frac{\partial^2}{\partial x^2} - \frac{\partial^2}{\partial y^2}\right)R_\sigma(r).$$

Thus, it can be considered as a linear combination of the 1D Laplace filter in the z direction and the 2D Laplace filter in the xy plane. Since the Laplace filter tends to emphasize the local changes of the electrostatic potential, enhanced spatial resolution is expected with a d_{z^2} tip.

Such an effect can be seen very clearly in electrostatic force (F_z) between a point charge (as a test) and different tips (Supplementary Fig. 3b). From the x-profile of F_z (Supplementary Fig. 3c), it is obvious that the peak width at half height with a d_{z^2} tip is much smaller than that with an s tip or a p_z tip. Besides, a "Mexican hat" shape can be seen at close distance (z=3 Å), which is also consistent with the DFT calculations in Supplementary Fig. 6. Therefore, the d_{z^2} tip does show higher spatial resolution compared with the s tip and p_z tip.

In the simulations of the CO-tip, we adopted σ = 0.7 Å and Q = -0.2 e, which means the quadrupole moment is $Q\sigma^2$= -0.20×0.5 e · Å^2 = -0.1 e · Å^2 = -0.48 Buckingham.

Supplementary Note 4. Effect of the stiffness (k) and charge (Q) on the simulated AFM images

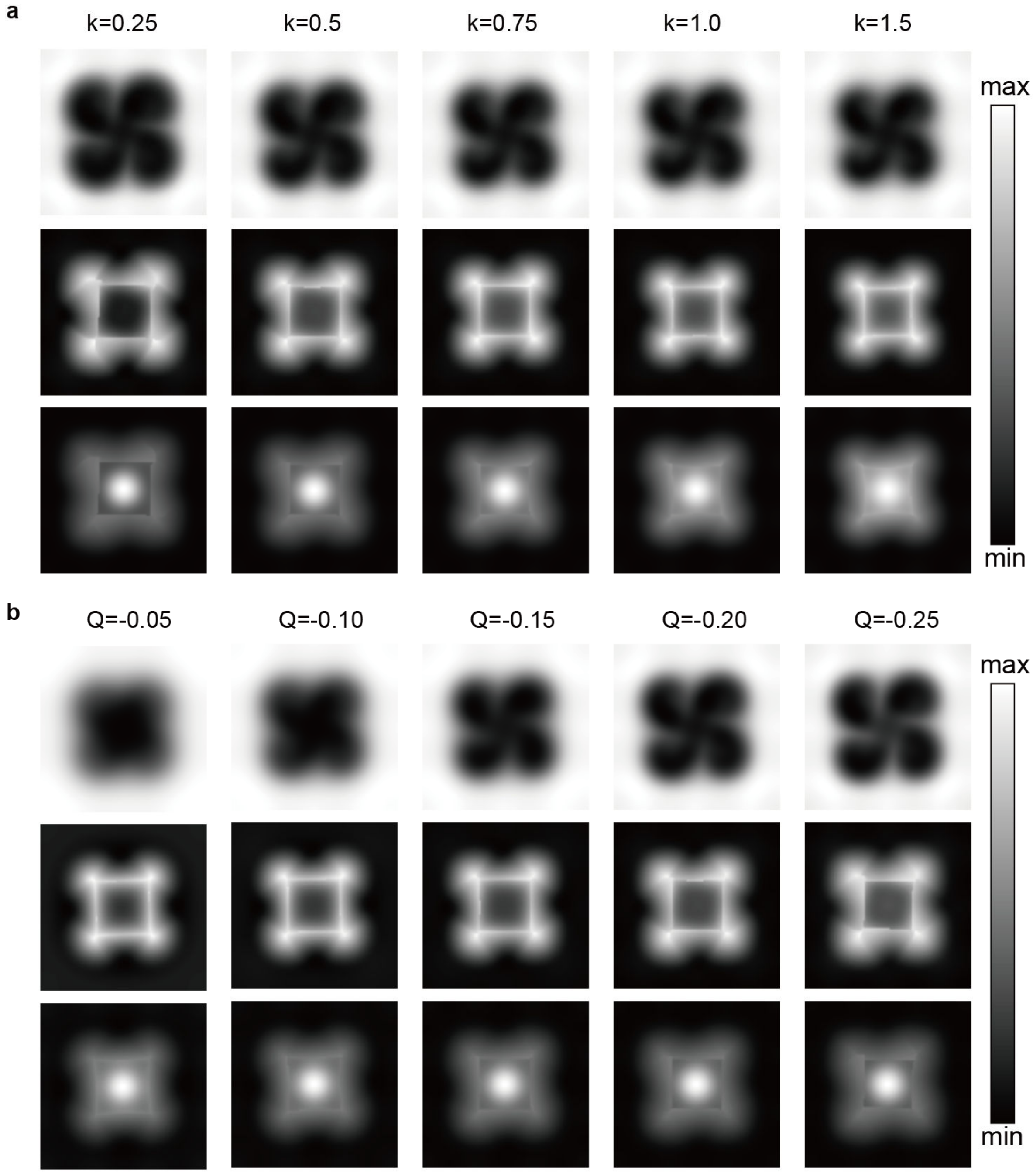

Supplementary Figure 4. The effect of the stiffness (k) and charge (Q) on the simulated AFM images of a water tetramer with a quadrupole (d_{z^2}) tip at different tip heights. (**a**) The effect of the stiffness (k) on the simulated Δf images. (**b**) The effect of the charge (Q) on

the simulated Δf images. The tip heights of the first, second and third rows are about 7.8 Å, 6.7 Å and 6.2 Å, respectively. For a better comparison, we had chosen similar simulation images by subtracting a small offset of tip height for different tips. The tip height in simulations is defined as the distance between the tip apex and the outmost H atom of water tetramer. All the oscillation amplitudes are 100 pm. The size of the images is 1.2 nm× 1.2 nm.

As the lateral stiffness (k) of the tip increases from 0.25 N m^{-1} to 1.5 N m^{-1}, the main features of AFM images do not change too much except for a slight distortion of the square at small tip height (Supplementary Fig. 4a, bottom), indicating the robustness of our simulation model. The best match between the experiment and the simulation was achieved with k=0.5 N m^{-1}. The stiffness 0.5 N m^{-1} adopted in the AFM is still well within the range reported for different metallic tips based on DFT simulations[3]. It should be noted that additional vertical force which is not included in the simulation (e.g. from background) could cause apparently larger value of lateral stiffness. When the effective charge Q varies from -0.05 e to -0.25 e, the AFM images at the large tip height show an improved spatial resolution (Supplementary Fig. 4b, top), suggesting that the electrostatic force plays a key role in the AFM imaging of the tetramer. The simulated images with Q from -0.15 e to -0.2 e match the experimental results the best (Supplementary Fig. 1f and k). At small tip heights, the effective charge has little effect on the contrast of the images due to the dominant role of Pauli repulsion (Supplementary Fig. 4b, middle and bottom). We notice that the sharp square shrinks a lot with larger Q due to the lateral relaxation of the probe particle induced by the electrostatic force.

Supplementary Note 5. Decay length of the force curves with different tips

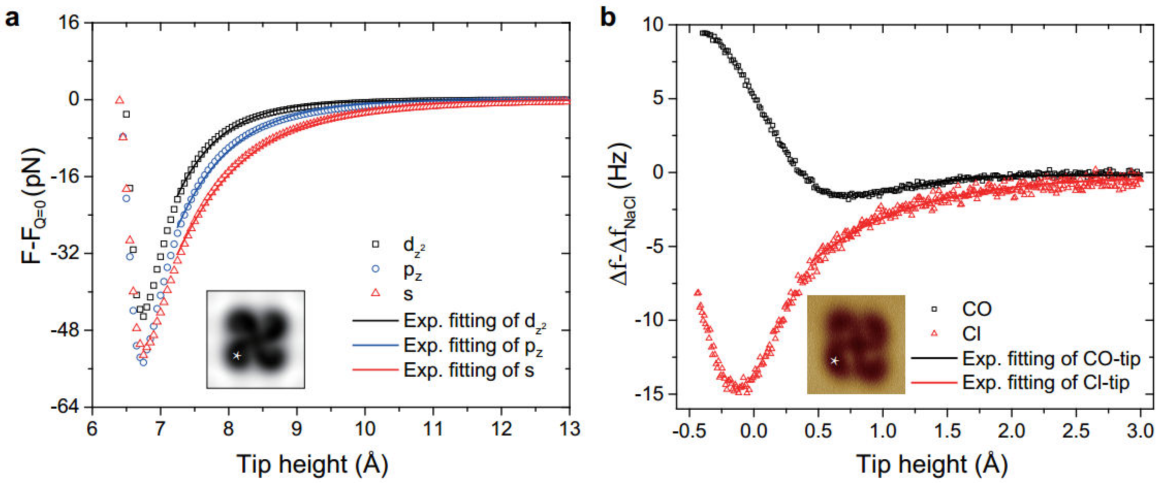

Supplementary Figure 5. The force curves between the water tetramer and different tips. (**a**) The calculated force curves with s, p_z and d_{z^2} tips after subtraction of the force with a neutral tip. The tip position is indicated with a star in the inset. The solid lines are the corresponding exponential fittings of the curves within the range of tip height from 7.2 Å to 13 Å. The tip height is defined as the distance between the tip apex and the outmost H atom of water tetramer. A high simulation cell (4 nm) was used here in order to accommodate realistic decay of electrostatic field. (**b**) The frequency shift Δf measured above the water tetramer (as indicated with a star in the inset) with CO- and Cl-tips after removing the contribution from the NaCl substrate. The solid lines are the corresponding exponential fittings of the curves within the range of tip height from 0.45 Å (Cl-tip) or 1.25 Å (CO-tip) to 3 Å. The tip height is with respect to the set point of 100 mV and 50 pA on NaCl. The decay lengths of different tips are summarized in Supplementary Table 1.

To extract the contribution of electrostatic force, we plotted the calculated force curves with s, p_z and d_{z^2} tips after subtraction of the force with a neutral tip (Supplementary Fig. 5a). Approximatively, we used an exponential fitting to obtain the decay length of the electrostatic force between the tetramer and different tips. To avoid the effect of tip relaxation at short tip-water separation, only the data points at large tip heights were fitted. The decay length of the d_{z^2} tip is the smallest, as shown in Supplementary Table 1. Similarly, we exponentially fitted the experimental Δf curves with CO- and Cl-tips after removing the contribution from the NaCl substrate (Supplementary Fig. 5b). We found that the decay

length with the Cl-tip is more than two times larger than that with the CO-tip (Supplementary Table 1), indicating the short-range nature of the high-order electrostatic force between the CO-tip and the water molecules.

Supplementary Note 6. Electrostatic field of Cl-tip vs CO-tip

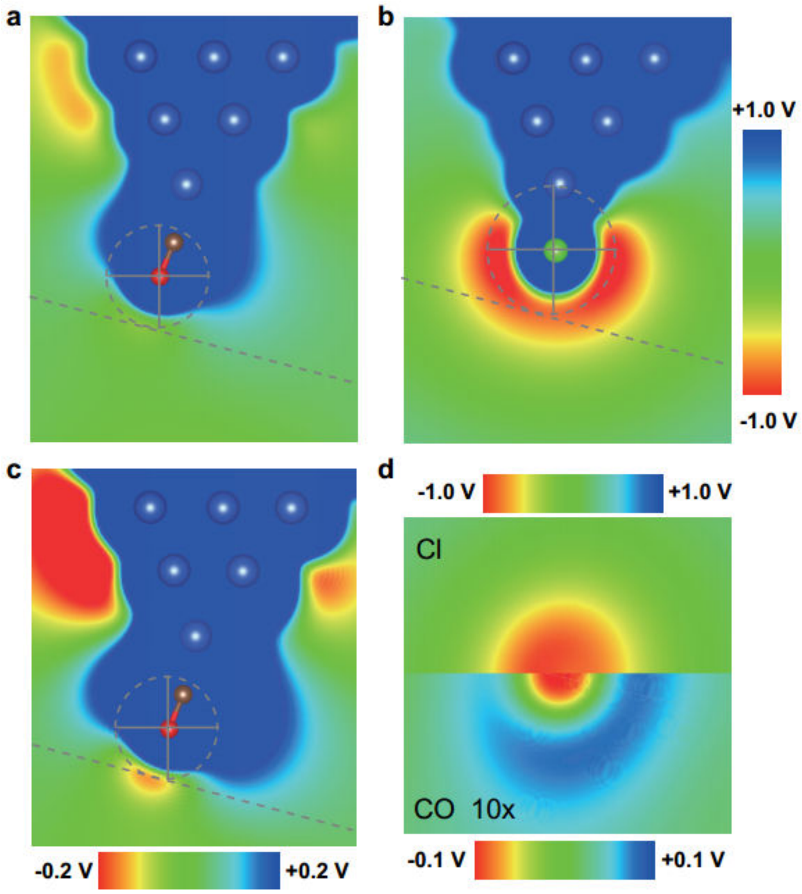

Supplementary Figure 6. Electrostatic field of Cl-tip vs CO-tip. (**a** and **b**) xz-cut of Hartree potential of CO (**a**) and Cl (**b**) functionalized tip plotted in the same range (+/- 1.0V) obtained from the total energy DFT simulations. Gray dotted circle denotes the van der Waals radius of the apex atom, Cl and O, respectively. The dark blue area around the tip atoms is due to unscreened potential of nuclei. Only the potential outside the vdW radius is relevant. (**c**) Hartree potential of the CO-tip plotted in a finer range +/-0.2V revealing a small negative cup below the oxygen atom. (**d**) Top view of the electrostatic potential (cut planes along the gray dotted lines shown in (**a**) and (**b**)) comparing Cl- and CO-terminated tips.

For comparison, we mapped the electrostatic field distribution of Cl-tip and CO-tip by DFT calculations (Supplementary Fig. 6a, b and c). As shown in Supplementary Fig. 6d, although the Cl-tip (upper half) has much stronger electrostatic field, the quadrupole-like CO-tip (lower half) has a highly localized negative potential at the CO apex showing a "Mexican hat" wavelet-like profile, which is quite similar to the Laplacian of Gaussian function. Thus, the CO-tip indeed behaves as a high-pass filter which can further enhance the spatial resolution by removing the slowly changed background. All these features agree quite well with that of the d_{z^2} tip model (see Supplementary Fig. 3).

Supplementary Note 7. Effect of the oscillation amplitude on Δf images

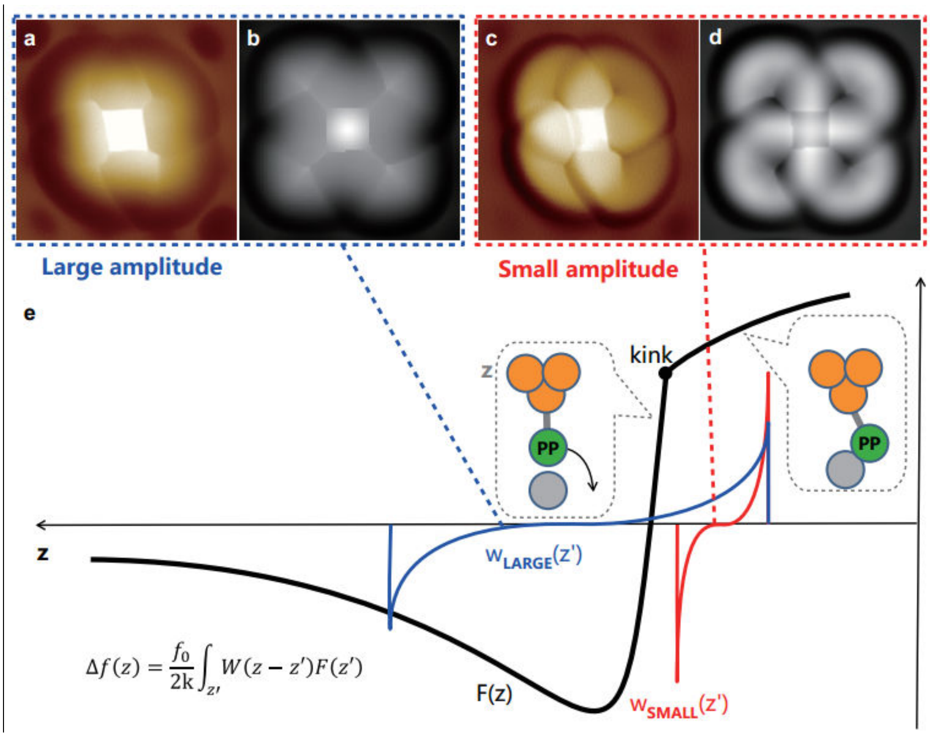

Supplementary Figure 7. Contrast variation in Δf images with oscillation amplitude. (**a** and **b**) Experimental (**a**) and simulated (**b**) Δf images obtained with large oscillation amplitude (100 pm). (**c** and **d**) Experimental (**c**) and simulated (**d**) Δf image obtained with small oscillation amplitude (40 pm). (**b**) and (**d**) were obtained from the same simulated force data, using just different amplitude parameters in force-to-Δf conversion procedure. (**e**) Schematic diagram showing force vs. distance curve and weighting function *w* for small (red) and large (blue) oscillation amplitude[4]. The kink of the force curve is due to sudden lateral

relaxation of the probe particle when lateral component of Pauli repulsion overcomes restoring spring force.

From the comparison of AFM images acquired with the Cl-tip using large (Supplementary Fig. 7a and b) and small amplitude (Supplementary Fig. 7c and d), it is evident that the small-amplitude regime is much more sensitive to the chiral shape of the electrostatic potential. In general, the Δf signal results from a weighted convolution of the force over a range of the oscillation amplitude[4]. In the case of large oscillation amplitude, the probe spends large part of the oscillation period at tip-sample distances, where the chirality of the electrostatic potential is almost negligible (Supplementary Fig. 7e, blue curve). In addition, the electrostatic potential changes significantly at the very close distance, having a non-trivial 3D chiral character. In the limit of the small amplitude, the frequency shift is proportional to derivative of force along z-distance (Supplementary Fig. 7e, red curve). Therefore, the non-trivial 3D character of the electrostatic potential induces a significant impact on the frequency shift when small amplitude is used. This chirality is further enhanced by a contrast inversion of the sharp features in AFM images (Supplementary Fig. 7a-d), which is caused by a sudden lowering of the slope of the force curve when the probe particle is deflected laterally (Supplementary Fig. 7e).

Supplementary Note 8. The relaxation of different tip apex in the AFM imaging

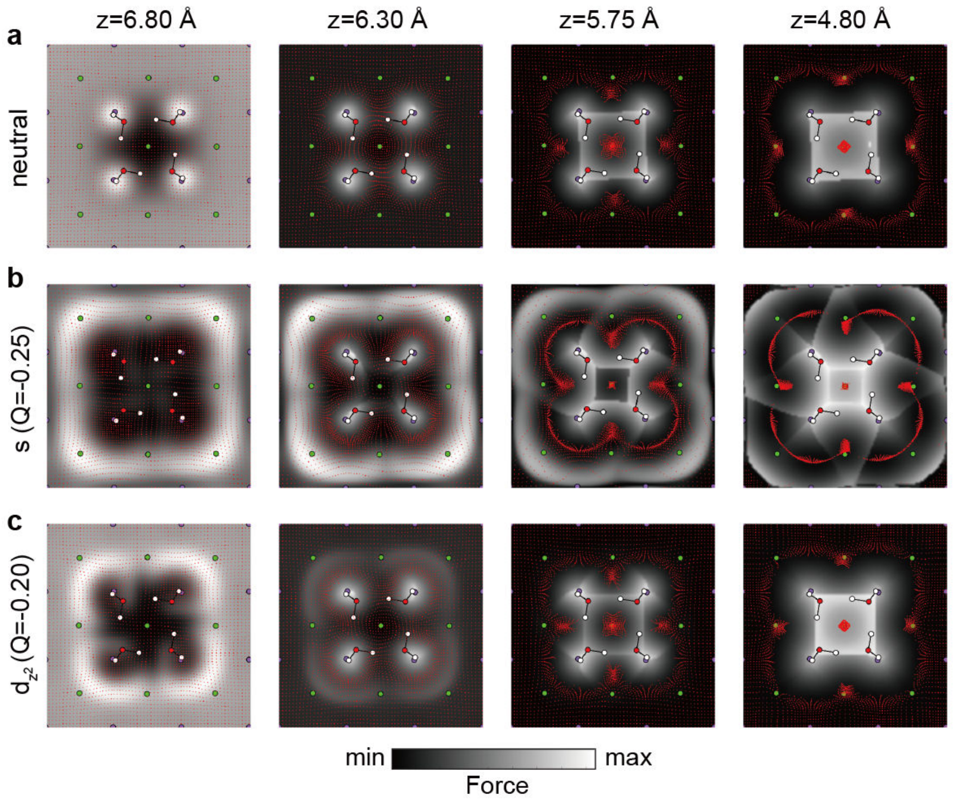

Supplementary Figure 8. The relaxation of different tip apexes in the AFM imaging. (**a**-**c**) Two-dimensional maps of the calculated vertical deflection (grey-scale background) and lateral relaxation (red dots) of the probe particle, respectively. The vertical deflection is related to the vertical force by Hooks law. The schematic tetramer structure and the underneath NaCl lattice are superimposed in the maps. H, O, Na, Cl atoms are denoted as white, red, purple and green spheres, respectively. (**a**), (**b**) and (**c**) were obtained with neutral (Q = 0), s (Q = -0.25 e) and d_{z^2} (Q = -0.2 e) tips, respectively. The stiffness (k) of the tips is 0.5 N m^{-1}. The definition of the tip height z is the same as in Supplementary Fig. 3. The size of all the images is 1.2 nm× 1.2 nm.

As the tip height decreases, the interaction between the tip and the tetramer induces significant lateral tip relaxation for all tip models (Supplementary Fig. 8). The neutral and d_{z^2} tips only deflect just over the dangling OH due to the Pauli repulsion force (Supplementary Fig. 8a and c), while the s-like tip apex surfs on the isosurface of the Pauli potential and meanwhile is strongly modulated by the attraction/repulsion of electrostatic

force, giving rise to the chiral features resembling the electrostatic potential distribution (Supplementary Fig. 8b). In contrast, at the large tip height where the chiral electrostatic potential of tetramer is resolved with the d_{z^2} tip, the lateral relaxation of the tip apex is negligible (Supplementary Fig. 8c, z=6.8 Å).

Supplementary Note 9. Calculated electrostatic potential map of the water dimers and trimers

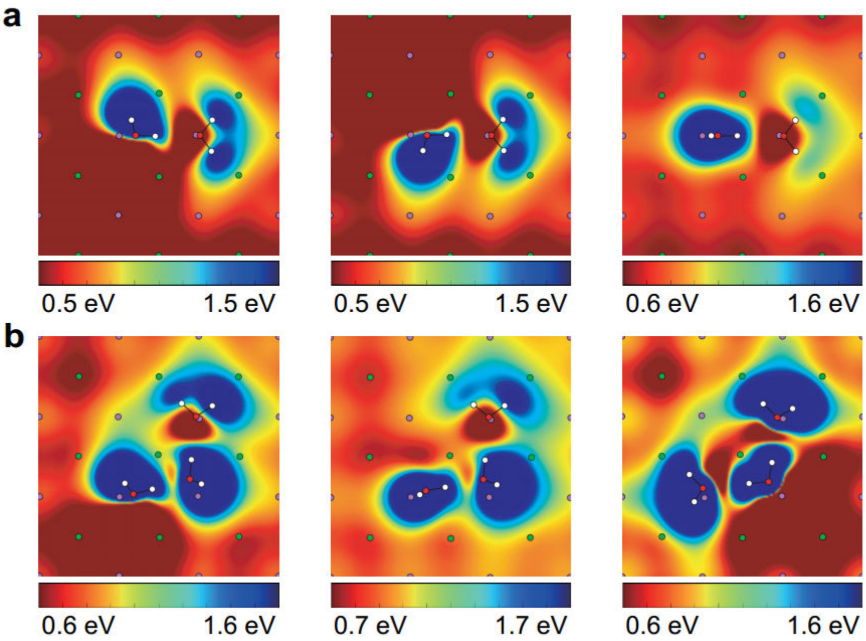

Supplementary Figure 9. Calculated electrostatic potential map of the water dimers and trimers. (**a**) Three water dimers corresponding to the ones in Fig. 4a. (**b**) Three water trimers corresponding to the ones in Fig. 4e. The plane height (which is defined as the distance from the outermost H atom) of the maps: (**a**) 45 pm, 45 pm, 49 pm (from left to right); (**b**) 23 pm, 30 pm, 14 pm (from left to right). H, O, Cl and Na atoms are denoted as white, red, green and purple dots, respectively. The size of all the images is 1.2 nm× 1.2 nm. The blue regions in the electrostatic potential maps arise from the positively charged H. From the characteristic shape and contrast of the blue features, we can easily determine the orientation of water molecules.

Supplementary Note 10. Tip-induced switching of water dimers and trimers

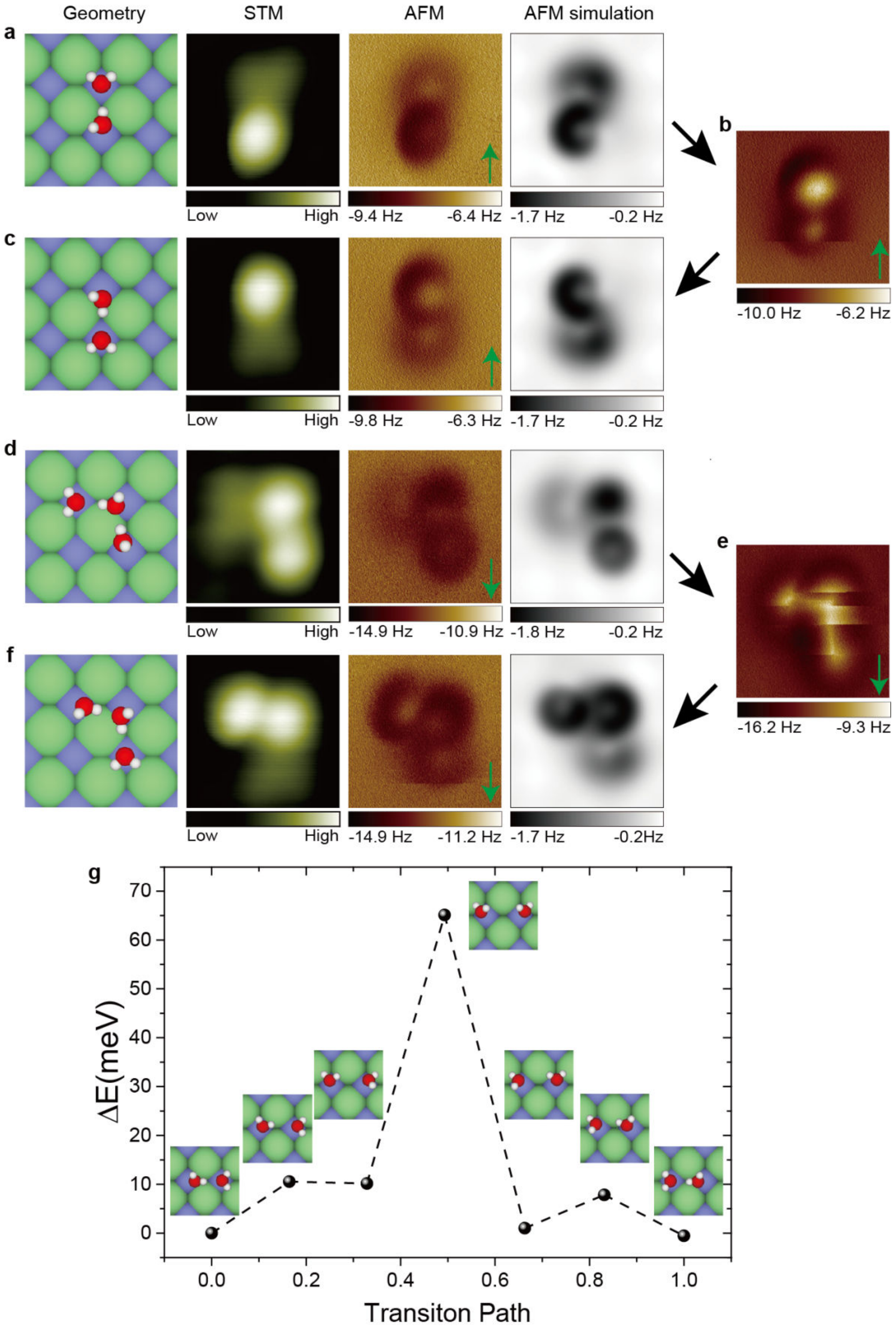

Supplementary Figure 10. Tip-induced switching of a water dimer and trimer. (a/d) and

(**c**/**f**) Geometric structures, STM image, experimental and simulated Δf image of a water dimer/trimer before (**a**/**d**) and after (**c**/**f**) the switching, respectively. (**b**/**e**) Closer imaging induced switching of the water dimer/trimer. The STM/AFM images of the dimer (or trimer) before and after the transition were obtained with the same CO-tip. The green arrows in the experimental AFM images indicated the scanning direction. Set point of STM images: (**a**,**c**) 100 mV and 30 pA; (**d**,**f**) 100 mV and 20 pA. The tip height of experimental AFM images: (**a**, **b** and **c**) 120 pm, 70 pm and 110 pm; (**d**, **e** and **f**) 130 pm, 30 pm and 110 pm. All the oscillation amplitudes of experimental and simulated images are 100 pm. All the simulations were done with a quadrupole (d_{z^2}) tip (k= 0.5 N m^{-1}, Q= -0.2 e). The size of the images is 1.2×1.2 nm^2. (**g**) Donor-acceptor exchanging barrier of the water dimer.

It turns out that the three different orientations of the water dimer (Fig. 4a) cannot be switched between each other, since they may have different adsorption energies at a particular site on the surface, leading to the selective stabilization of one of those dimers at different sites. The adsorption inhomogeneity mainly arises from the herringbone reconstruction of the underlying Au(111) substrate, which breaks the energetic degeneracy and inhibits the switching. Instead, we found that the water dimer and trimer can be switched through a H-bond donor-acceptor exchanging process (Supplementary Fig. 10). Such a switching can be easily induced by CO-tips at small tip heights, but we were able to achieve submolecular-resolution AFM imaging at relatively large tip heights, thus allowing the accurate assignment of those weakly bonded clusters before and after the switching. With Cl-tips, it is very difficult to maintain stable STM/AFM imaging even at large tip heights due to the strong electrostatic interaction between the Cl-tip and the water molecules (Supplementary Fig. 12). The switching of the dimer and trimer recorded with the same CO-tips at the same adsorption sites indicates that the contrast observed in AFM images does not arise from the asymmetry/scanning directions of the tip nor the different adsorption sites, but depends on the orientation of the water molecules. In fact, these crooked depressions in the AFM images are correlated with the position of the H atoms, which is corroborated by the AFM simulations based on the geometric structure through DFT calculations.

Supplementary Note 11. The vertical and lateral forces needed to obtain submolecular resolution

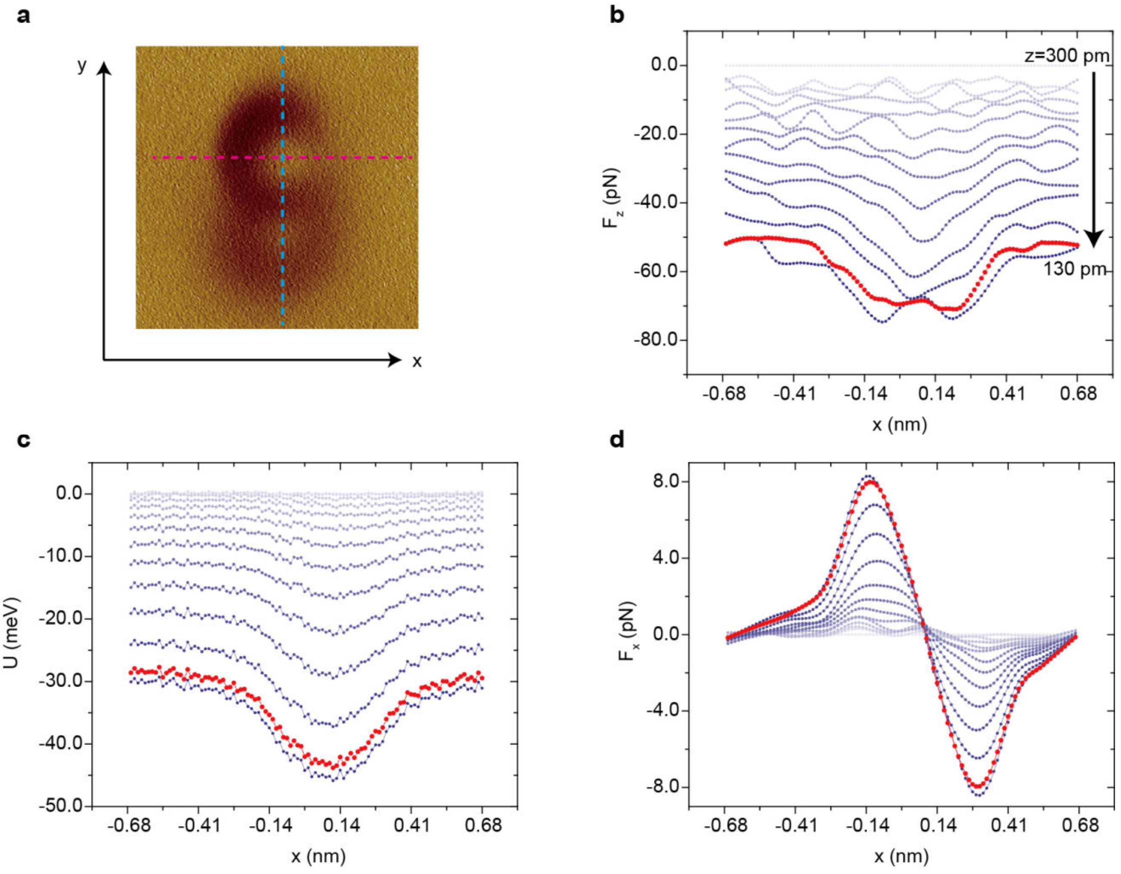

Supplementary Figure 11. The vertical and lateral force needed to obtain submolecular resolution of a water dimer. (a) Dashed red (blue) line shows the x (y) direction along which the frequency shift data at different tip heights (z=300 pm to 130 pm) were obtained. Note that the frequency shifts are related to the weighted average of interaction force over the cantilever oscillation between z=z' and z=z'+2A. The amplitude A is 100 pm. (**b** to **d**) Vertical force F_z, tip-sample interaction energy U, and lateral force F_x extracted from the frequency shift along x direction. The tip height changes every 5 pm (only some of them are shown here). The red curves in (**b-d**) correspond to the tip height where the submolecular resolution can be obtained. We also carried out similar measurements along the dashed blue line (y direction) as shown in (**a**). The corresponding lateral force and interaction energy needed to obtain submolecular resolution are 10.5 pN and 56 meV, respectively.

In order to be more quantitative on how "weak" the perturbation of AFM imaging can be, we performed systematic AFM measurements to estimate the minimum forces needed to achieve the submolecular resolution, following the method described in Supplementary ref. 5. We can get F_z and the interaction energy U from the frequency shift Δf at different tip heights according to Supplementary ref. 6 (Supplementary Fig. 11). The lateral force F_x (F_y) can be then obtained from the partial differentiation of the interaction energy U along x (y) direction. It can be seen that the minimum vertical (z) force which yields the submolecular resolution of the water dimer is only about 70.9 pN. The minimum lateral force along x (y) direction is 7.9 (10.5) pN. The corresponding tip-water interaction energy is as small as 40-50 meV, which allows the imaging of metastable water structures with very small transition barrier (Supplementary Fig. 10).

Supplementary Note 12. The disturbance of Cl-tip on the water dimer, trimer and double-tetramer

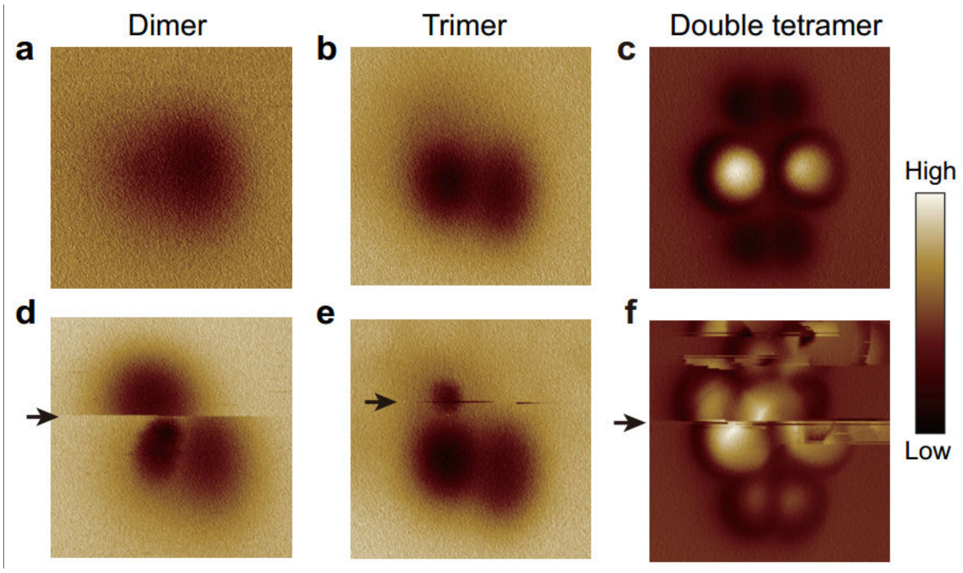

Supplementary Figure 12. The disturbance of Cl-tip on the water dimer, trimer and double-tetramer. (**a** and **d**) Δf images of a water dimer at tip heights of 140 pm and 120 pm, respectively. (**b** and **e**) Δf images of a water trimer at tip heights of 0 pm and -30pm, respectively. (**c** and **f**) Δf images of a water double-tetramer at tip heights of -50pm and -100pm, respectively. Similar to the structure of triple tetramer (Supplementary Fig. 10), the

double tetramer consists of two tetramers linked by two bridged water molecules. The tip height is referenced to the STM set point on the NaCl surface (100 mV, 50 pA). The oscillation amplitudes: (**a**), (**b**), (**d**) and (**e**) 100pm; (**c**) and (**f**) 50pm. The size of the images: (**a**), (**b**), (**d**) and (**e**) 1.4 nm× 1.4 nm; (**c**) and (**f**) 2 nm× 2 nm.

Supplementary Table 1 The fitted decay length of force curves obtained with different tips

Calculated decay length (Å)			Experimental decay length (Å)	
s tip	p_z tip	d_{z^2} tip	Cl-tip	CO-tip
1.039±0.006	0.814±0.005	0.654±0.004	0.786±0.014	0.326±0.03

Supplementary Table 2 Parameters of Lennard Jones pairwise potentials for all elements

Element	ε [meV]	r [Å]
H	0.680	1.487
O	9.106	1.661
Cl	11.491	1.948
Na	10.0	1.4

Supplementary Table 3 | The adsorption energies of those metastable water trimmers

Trimer	Fig. 4e (left)	Fig. 4e (middle)	Fig. 4e (right)
E_{ads} (eV)	1.618	1.607	1.571

Supplementary References

1. Hapala, P. *et al.* Mechanism of high-resolution STM/AFM imaging with functionalized tips. *Phys. Rev. B* **90**, 085421 (2014).
2. Richmond, T. J. Solvent accessible surface area and excluded volume in proteins: Analytical equations for overlapping spheres and implications for the hydrophobic effect. *J. Mol. Biol.* **178**, 63-89 (1984).
3. Gross, L. *et al.* Bond-Order Discrimination by Atomic Force Microscopy. *Science* **337**, 1326-1329 (2012).
4. Giessibl, F. J. A direct method to calculate tip-sample forces from frequency shifts in frequency-modulation atomic force microscopy. *Appl. Phys. Lett.* **78**, 123-125 (2001).
5. Ternes, M. *et al.* The force needed to move an atom on a surface. *Science* **319**, 1066-1069 (2008).
6. Sader, J. E. & Jarvis, S. P. Accurate formulas for interaction force and energy in frequency modulation force spectroscopy. *Appl. Phys. Lett.* **84**, 1801-1803 (2004).
